# Nonhemodynamically Significant Coarctation of Aorta: Is it Clinically Significant?∗

**DOI:** 10.1016/j.jacadv.2023.100674

**Published:** 2023-10-25

**Authors:** Alexander C. Egbe, Heidi M. Connolly

**Affiliations:** Department of Cardiovascular Medicine, Mayo Clinic Rochester, Rochester, Minnesota, USA

**Keywords:** coarctation of aorta, exercise capacity, left ventricular afterload

**Figure:**
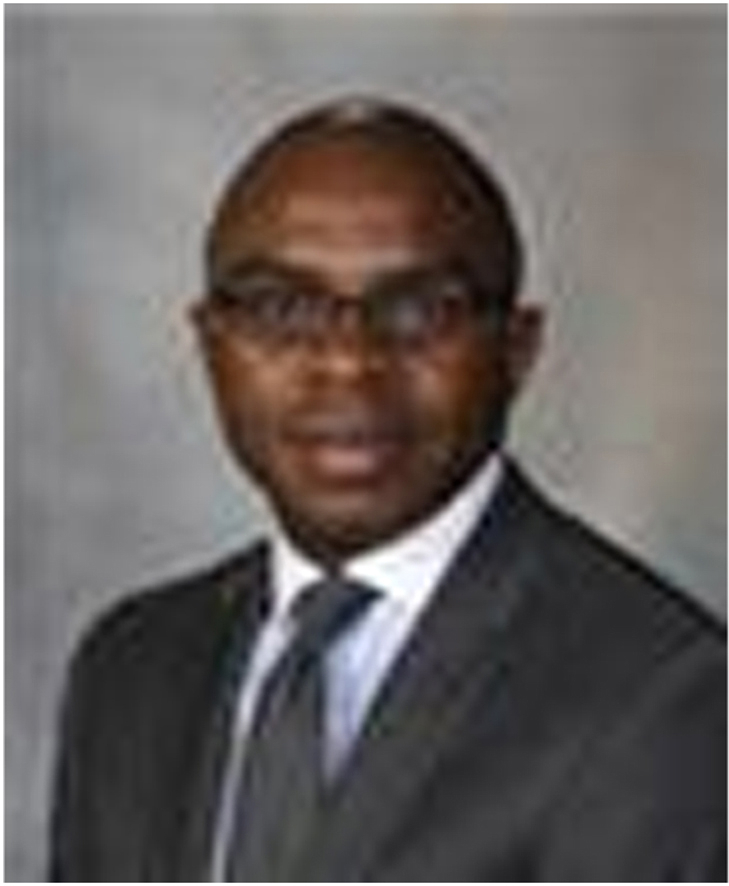

We congratulate Ramachandran et al^1^ for their recent article titled ‘Anatomical/Physiological Correlates of Functional Capacity in Adults with Repaired and Non-Severe Coarctation of the Aorta.’ This was a retrospective cross-sectional study assessing the relationship between the severity of coarctation of aorta (COA) and exercise capacity in patients without hemodynamically significant COA, albeit, limited by small sample size. The study showed an association between COA severity, as measured by aortic isthmus gradient and aortic isthmus size, and exercise capacity, as measured by exercise time and metabolic equivalents (METs).

COA is characterized by aortic isthmus stenosis and/or aortic arch hypoplasia.^2^^,^^3^ In addition to these anatomic defects, patients with COA also have physiologic defects characterized by endothelial dysfunction, abnormal smooth muscle reactivity, and abnormal sympathetic activation, and these in turn, lead to increased aortic stiffness.^4^ In addition to these anatomic and physiologic defects, the differences in tissue characteristics of the aortic isthmus, especially in patients with prior COA repair, alter aortic dynamics leading to early arrival of the reflected aortic wave in late systole (instead of early diastole).^5^ This leads to an increase in the left ventricular (LV) systolic afterload, as well as a decrease in diastolic perfusion pressure required for adequate coronary blood flow and myocardial perfusion.^5^^,^^6^ Collectively, these anatomic and physiologic abnormalities lead to LV remodeling, that is characterized by LV hypertrophy, diastolic dysfunction (impaired relaxation and compliance), and systolic dysfunction.^5^^,^^7^^,^^8^

Transcatheter and surgical COA repair have been shown to be effective in relieving the anatomic defect associated with COA.^9^^,^^10^ However, these interventions are associated with procedural complications, as well as need for future reinterventions because of recurrent COA.^11^^,^^12^ In order to maximize the benefits and minimize the risks associated with COA repair, the American and European guidelines for the management of adults with congenital heart disease recommend surgical or transcatheter COA repair for patients with ‘hemodynamically significant COA.’^11^^,^^12^ The definition of ‘hemodynamically significant COA’ is quite variable and complicated, but it is generally accepted as COA Doppler mean gradient or peak-to-peak gradient >20 mm Hg.^11^^,^^12^ This cutoff point is used in clinical practice for deciding on the timing of COA intervention.

While the widespread adoption of these criteria for defining the hemodynamic significance of COA has been effective in streamlining timing and indications for COA intervention, it inadvertently creates a false sense of security for patients with COA gradient ≤20 mm Hg, as being in the ‘safe zone.’ The current study by Ramachandran et al demonstrated that, even within this ‘safe zone’ of COA gradient ≤20 mm Hg, there was a significant negative correlation between COA gradient and exercise capacity (exercise time and METs), suggesting that the ‘safe zone’ may not be very safe. The authors also showed that patients with coarctation-to-diaphragm ratio (also known as aortic isthmus ratio) >0.7 had lower exercise capacity as measured by exercise time and METs. Interestingly, while coarctation-to-diaphragm ratio >0.7 was an arbitrary cutoff point in this study, it has been empirically shown as the optimal cutoff point to predict LV remodeling (hypertrophy, diastolic, and systolic dysfunction), symptoms, and cardiovascular events in COA.^13^, ^14^, ^15^

Once again, we congratulate Ramachandran et al for demonstrating that, even within the subgroup of patients with nonhemodynamically significant COA, minimal increase in COA gradient had negative correlation with exercise capacity. This is quite concerning because impaired exercise capacity is a well-established negative prognostic marker in patients with cardiovascular disease. This study also raises important questions regarding the optimal diagnostic and management strategies for patients with “non-severe COA” and supports incorporating regular exercise testing in the assessment of patients with COA. However, we need better tools to determine which COA patients benefit from intervention even when the COA gradient or dimension is not at the current recommended threshold, whether intensification of medical therapy for afterload reduction would improve outcomes in this subset of COA patients and what type of intervention results in the best outcome.

## Funding support and author disclosures

Dr Egbe is supported by 10.13039/100000050National Heart, Lung, and Blood Institute (NHLBI) grants (R01 HL158517 and R01 HL160761). Dr Connolly has reported that she has no relationships relevant to the contents of this paper to disclose.
